# The Impact of the COVID-19 Pandemic on Medical Faculty’s Academic Performance and Well-Being: Short title: COVID-19 Pandemic and Medical Faculty’s Academic Performance and Well-Being

**DOI:** 10.31661/gmj.v15i.4139

**Published:** 2026-03-02

**Authors:** Asim Sharif

**Affiliations:** ^1^ Department of Medical Education, Faculty of Medicine, King Abdulaziz University, Jeddah, 21589, Saudi Arabia

**Keywords:** Anxiety, COVID-19, Depression, Generalized Anxiety Disorder-7, Patient Health Questionnaire-9

## Abstract

**Background:**

To investigate the effects of stress related to the pandemic on the medical faculty’s well-being.

**Materials and Methods:**

This study investigated the impact of pandemic-related stress on the well-being of medical faculty at King Abdulaziz University. Using a mixed-methods approach, 82 faculty members completed online surveys, including the GAD-7 and PHQ-9 scales to measure anxiety and depression, and participated in focus group discussions. The analysis explored the relationship between these psychological stress factors and self-reported academic performance across different demographics.

**Results:**

A high prevalence of psychological distress was found: 89.6% of faculty reported at least mild anxiety, and 84.4% reported at least mild depressive symptoms. Most participants self-rated their academic performance as good (54.5%) or excellent (36.4%), and 80.5% reported missing no crucial deadlines. Regression analyses revealed a significant positive association between depression severity and higher self-reported academic performance (β = 1.48, p = 0.025). Male gender (OR = 0.20, p = 0.022) and longer weekly working hours (OR = 0.53, p = 0.019) were associated with significantly lower odds of missing deadlines. Anxiety, depression, and other covariates were not significant predictors of overall quality of life.

**Conclusion:**

While the pandemic was associated with a high burden of anxiety and depression among faculty, this distress did not translate into widespread self-perceived declines in academic output. The counterintuitive link between higher depression scores and self-reported performance, alongside the protective effect of male gender and increased work hours against missed deadlines, suggests complex coping mechanisms and potential resilience or presenteeism.

## Introduction

COVID-19, known as the coronavirus, was first reported in 2019 and declared a global pandemic in March 2020 [1]. Because of this, governments around the world implemented lockdowns, travel bans, and school and university closures, which led to a swift transition to online education [2][3]. These shifts affected teaching and research in both tiers of academia [3].

It is said that because of the pandemic, global systems of higher education and health care were disrupted, causing significant effects on mental health and professional functioning of academic faculty [4][5]. Moreover, upon initiation of novel study, research, and clinical practices, there was a general surge in stress, anxiety, depression, and burnout, which was attributable to the transition [6].

According to a study done by Knapp et al., stress levels doubled between 2019 and 2020 [7]. Burnout, which was defined as emotional exhaustion, was on the rise amongst healthcare providers, which was a major concern to patients under their care [8]. In addition, there was a gradual decrement in work-life balance in 2020 [7]. This was mainly as a result of the transition from face-to-face to virtual learning. The staff reported they shifted gears towards virtual teaching, which greatly impacted research [9]. As a result, there was a 39% drop in published research papers [10].

The pandemic-related stress was not equally distributed across genders and age groups. Female staff and juniors notably experienced high levels of burnout and reduced productivity attributable to a juggle between work and household burdens [9][11][12]. A study by Knapp et al. revealed that 79% of females experienced burnout as compared to 59% of males [7].

However, there is limited knowledge on stress related to the pandemic amongst medical staff, hence creating a research gap. This study aims to fill this gap by looking into the level at which anxiety and depression affect faculty performance in their roles. Hence informs the requirements of sustaining mental as well as academic well-being.

## Methods and Study Design

### Study design

An amalgamation of study methods was employed. Cross-sectional surveys and semi-structured focus group discussions were used. The quantitative method was intended to characterize the extent and prevalence of anxiety and depression among clinical faculty members and to determine correlations with self-reported academic performance. The qualitative element pursued profound insights into the influence of COVID-19-related stress on faculty work, coping, and perceived institutional supports. The study was conducted in the Faculty of Medicine, King Abdulaziz University (KAU).

### Population and sampling

The target population comprised all faculty members in the clinical departments of KAU’s Faculty of Medicine, including Medicine, Surgery, Obstetrics & Gynecology, Pediatrics, ENT, Ophthalmology, Medical Education, Family Medicine, Community Medicine, Emergency Medicine, Urology, Dermatology, Orthopedics, Radiology, and Anesthesia. For the quantitative survey, all clinical faculty were invited to participate. For the qualitative component, purposive sampling was used to invite faculty who could provide in-depth information: six faculty members from the Internal Medicine department and four faculty members from the Family Medicine department were invited to take part in two Focus Group Discussions (total qualitative sample size = 10).

## Data collection instruments

### Quantitative instrument

A self-administered online questionnaire was deployed to all medical faculty. The instrument comprised of five domains: study introduction and informed consent; sociodemographic and work characteristics (age, gender, department, academic rank, years in position, weekly work hours); COVID-19-related work and personal struggles; perceptions of the pandemic’s impact on academic performance (self-rated performance, missed deadlines, perceived productivity and focus); and mental-health assessment (anxiety and depression). Anxiety was measured using the Generalized Anxiety Disorder-7 (GAD-7) scale and depression using the Patient Health Questionnaire-9 (PHQ-9). Both scales were scored using standard severity categories (minimal, mild, moderate, moderately severe, severe).

### Qualitative instrument

A semi-structured focus group discussion guide followed the questionnaire. Topics covered were: perceived implications of COVID-19 on teaching, research, and administrative responsibilities, specific stressors and coping mechanisms; perceptions of deadlines and their effects on productivity; and institutional support recommendations. The Focus Group Discussions were conducted using the same core questions by two trained moderators, who could probe divergent responses.

## Ethical considerations

The Biomedical Ethics Committee of the Faculty of Medicine, King Abdulaziz University, approved the study protocol, Approval No 237-21. Participation was out of free will, and informed consent was obtained before the data collection.

## Data Analysis

All quantitative data were analyzed using STATA version 17.0 (StataCorp, College Station, TX, USA). Descriptive statistics were used to summarize participant characteristics, work profiles, and mental health status. Continuous variables were reported as mean ± standard deviation (SD), and categorical variables were reported as frequencies and percentages. To examine associations between mental health variables and work-related outcomes, multivariable regression analyses were performed. Specifically, ordinal logistic regression was used to model self-reported academic performance, binary logistic regression was used to model the likelihood of missing crucial deadlines due to stress or mental health challenges, and multiple linear regression was used to model overall quality of life. All regression models were adjusted for key demographic and work-related covariates: gender, age, years at current position, and weekly working hours. Regression coefficients (b), standard errors (SE), odds ratios (OR), 95% confidence intervals (CI), and corresponding p-values were reported.

## Participant Characteristics

**Table:** Table1. Sociodemographic Characteristics, Work Profile, and Mental Health Status of Participants (N = 77)

**Variable**	**n (%) or Mean ± SD**
**Age (years)**	42.5 ± 8.1
**Years at current position**	3.8 ± 2.6
**Weekly working hours**	44.8 ± 1.7
	
**Gender**	
Male	46 (59.7)
Female	31 (40.3)
	
**Department**	
Medicine	37 (48.1)
Family Medicine	14 (18.2)
Medical Education	10 (13.0)
Others *	16 (20.7)
**Anxiety severity (GAD-7)**	
Minimal anxiety	8 (10.4)
Mild anxiety	21 (27.3)
Moderate anxiety	36 (46.8)
Severe anxiety	12 (15.6)
	
**Depression severity (PHQ-9)**	
Minimal depression	12 (15.6)
Mild depression	18 (23.4)
Moderate depression	30 (39.0)
Moderately severe depression	9 (11.7)
Severe depression	8 (10.4)

**Table:** Table2. Outcome Comparisons by Age Group and Gender (N = 77)

**Group / Outcome**	**Academic Performance**	**Missed Deadlines**	**Anxiety**	**Depression**	**Qol**
Age					
30-39	2.35 ± 0.65	0.15 ± 0.36	2.85 ± 0.86	3.06 ± 1.20	2.50 ± 0.93
40-49	2.13 ± 0.63	0.23 ± 0.43	2.33 ± 0.84	2.30 ± 1.09	2.60 ± 0.97
50-59	2.33 ± 0.50	0.22 ± 0.44	2.89 ± 0.78	3.00 ± 0.87	2.89 ± 1.27
60+	2.50 ± 0.58	0.25 ± 0.50	3.25 ± 0.50	3.50 ± 1.00	2.50 ± 1.29
p-value (age)	0.443	0.829	0.031	0.022	0.746
Gender					
Male	2.26 ± 0.57	0.11 ± 0.31	2.76 ± 0.87	2.91 ± 1.15	2.57 ± 1.09
Female	2.29 ± 0.69	0.32 ± 0.48	2.55 ± 0.85	2.58 ± 1.18	2.61 ± 0.84
p-value (gender)	0.717	0.021	0.304	0.228	0.837

**Qol:** quality of life

A total of 77 clinical faculty members participated in the quantitative survey. The mean age of participants was 42.5 ± 8.1 years (range: 30-65 years). On average, participants had been in their current academic position for 3.8 ± 2.6 years, with a median of 3 years. Faculty members reported working a mean of 44.8 ± 1.7 hours per week, with most participants working approximately 45 hours weekly.

Male participants constituted 59.7% (n = 46) of the sample, while 40.3% (n = 31) were female. Participants were drawn from a variety of clinical departments, with the largest representation from the Department of Medicine (48.1%), followed by Family Medicine (18.2%) and Medical Education (13.0%). Other departments each accounted for smaller proportions of the sample.

Symptoms of anxiety were highly prevalent among faculty members. Nearly half of participants (46.8%) reported moderate anxiety, while 27.3% reported mild anxiety. Severe anxiety was reported by 15.6% of respondents, whereas only 10.4% reported minimal anxiety symptoms.

Similarly, depressive symptoms were common. Moderate depression was reported by 39.0% of participants, followed by mild depression (23.4%). Minimal depression was reported by 15.6%, while 11.7% experienced moderately severe depression and 10.4% reported severe depression. Overall, more than two-thirds of respondents reported at least mild depressive symptoms (Table-1).

Data are presented as mean ± standard deviation or number (percentage). Anxiety severity was assessed using the Generalized Anxiety Disorder-7 (GAD-7) scale and depression severity using the Patient Health Questionnaire-9 (PHQ-9). * Surgery (n=5), Pharmacology (n=4), Medical Genetics (n=2), Microbiology & Parasitology, Obstetrics & Gynecology, and ENT each one person.

Table-2 summarizes outcomes stratified by age group and gender. The distribution of self-reported academic performance did not differ significantly between male and female faculty members (p = 0.348) or across age groups (p = 0.577). Regarding missed deadlines, female faculty reported missing deadlines more frequently than males, with the difference reaching statistical significance (p = 0.042), although no significant differences were observed across age groups (p = 0.829). Anxiety severity differed by age group (p = 0.036) but not by gender (p = 0.667), whereas depressive symptoms showed borderline significance across age groups (p = 0.050) and no gender differences (p = 0.222). Overall quality of life did not significantly differ by gender (p = 0.151) or age group (p = 0.899).

## Result

**Table:** Table3. Multivariable Regression Models Examining Academic Performance, Missed Deadlines, and Quality of Life (N = 77)

**Predictor**	**Academic Performance (Ordinal Logistic Regression)**	**Missed Deadlines (Binary Logistic Regression)**	**Quality of Life (Linear Regression)**
	*b* (SE)	OR (95% CI)	*b* (SE)
Anxiety severity	−0.04 (0.83), p = .960	6.25 (0.66-59.63), p = .111	−0.24 (0.31), p = .456
Depression severity	1.48 (0.66), p = .025	0.23 (0.04-1.32), p = .099	0.10 (0.23), p = .670
Gender (male)	−0.67 (0.53), p = .210	0.20 (0.05-0.79), p = .022	0.24 (0.20), p = .239
Age (years)	0.00 (0.06), p = .947	0.92 (0.78-1.09), p = .330	0.04 (0.02), p = .131
Years at current position	−0.10 (0.17), p = .578	1.09 (0.72-1.65), p = .690	−0.03 (0.07), p = .624
Weekly working hours	−0.06 (0.19), p = .768	0.53 (0.31-0.90), p = .019	0.01 (0.08), p = .867

The study included 82 medical faculty members, of whom 46 (59.7%) were male and 36 (40.3%) were female. Participants had spent an average of 6.4 years in their current academic position. Nearly half of the respondents (45.5%) had either two years (23.4%) or five years (22.1%) of professional experience in their current role. Most participants reported working approximately 45 hours per week (66.2%).

Regarding academic performance, the majority of participants rated their performance as good (54.5%) or excellent (36.4%), whereas only 9.1% reported fair performance. In terms of work-related outcomes, 80.5% of faculty members indicated that they had not missed any important deadlines due to stress or mental health-related challenges; however, 19.5% reported missing at least one deadline.

**Figure-1:**
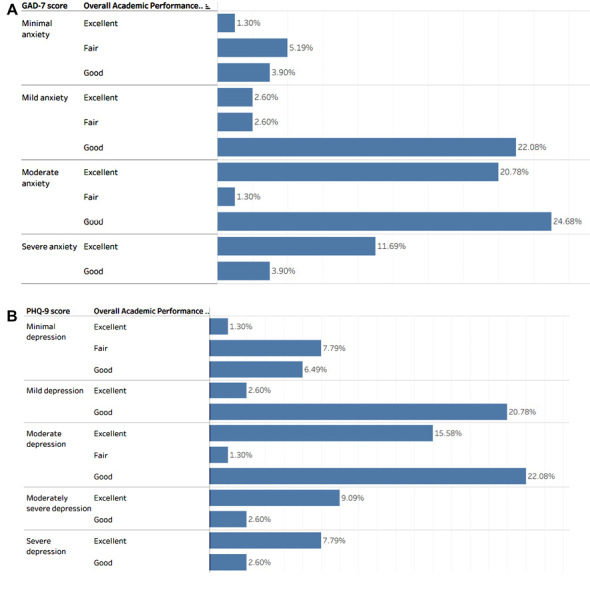
Figure 1. Distribution of anxiety categories based on GAD-7 scores (A) and depression severity based on PHQ-9 scores (B).

Figure 1 presents the distribution of anxiety and depression levels among participants based on GAD-7 and PHQ-9 scores, respectively. Moderate anxiety symptoms were observed in 46.8% of participants, indicating a considerable burden of psychological distress among medical faculty members. Similarly, depressive symptoms were prevalent, with 38.9% of respondents demonstrating moderate levels of depression.

### Regression analyses

Multivariable regression analyses were conducted to examine the associations between anxiety, depression, and work-related outcomes while adjusting for gender, age, years at current position, and weekly working hours in all models (Table-3).

Ordinal logistic regression was used to assess predictors of self-reported academic performance. After adjustment, depression severity was significantly associated with academic performance, with higher depression scores associated with increased odds of reporting higher academic performance (β = 1.48, SE = 0.66, p = 0.025). Anxiety severity was not significantly associated with academic performance (p = 0.960). Gender, age, years at current position, and weekly working hours were also not significantly associated with academic performance.

Binary logistic regression was performed to identify predictors of missing crucial deadlines due to stress, worry, or depression. The adjusted model was statistically significant (likelihood ratio test p = 0.026) and accounted for approximately 19% of the variance in missed deadlines (pseudo R² = 0.19). Male gender was associated with significantly lower odds of missing deadlines compared to female gender (OR = 0.20, 95% CI [0.05, 0.79], p = 0.022). Additionally, greater weekly working hours were associated with lower odds of missing deadlines (OR = 0.53, 95% CI [0.31, 0.90], p = 0.019). Anxiety severity showed a non-significant positive association (p = 0.111), while depression severity demonstrated a non-significant negative association (p = 0.099). Age and years at current position were not significantly associated with missed deadlines.

Multiple linear regression was conducted to examine predictors of overall quality of life. After adjustment for all covariates, the model was not statistically significant (F = 0.95, p = 0.468), and none of the included predictors were significantly associated with quality of life.

All models were adjusted for gender, age, years at current position, and weekly working hours. Academic performance was analyzed using ordinal logistic regression; missed deadlines were analyzed using binary logistic regression; and quality of life was analyzed using multiple linear regression. OR = odds ratio; CI = confidence interval.

## Discussion

This study aimed to examine the impact of anxiety and depression on academic performance and the ability to meet deadlines among clinical faculty members. Despite a high prevalence of mild to moderate depressive symptoms (62.4% reporting at least mild depression) and moderate anxiety (46.8%), academic performance was generally not adversely affected. This suggests a degree of resilience among faculty in maintaining academic productivity despite psychological distress.

Our GAD-7 and PHQ-9 outcomes indicate that a substantial proportion of faculty experienced moderate to severe mental health symptoms, reflecting an increased psychological burden, which aligns with previous studies by Sukhawathanakul et al. [13] and Yu et al. [14]. Interestingly, regression analyses revealed that higher depression severity was significantly associated with higher self-reported academic performance, while anxiety severity did not show a significant effect. This supports the idea that moderate psychological distress does not uniformly impair academic output and may, in some cases, coincide with heightened focus or compensatory efforts, as suggested by prior literature [15]. Weinreich et al. [16] similarly reported that productivity remained comparable across genders despite moderate stress levels, which aligns with our finding of no significant gender differences in academic performance.

Regarding work-related outcomes, 19.5% of faculty reported missing at least one crucial deadline, with female faculty more likely to miss deadlines than males (p = 0.042). Regression analyses further indicated that male gender and greater weekly working hours were associated with lower odds of missing deadlines, highlighting potential differences in coping strategies or workload management. These findings echo literature suggesting that anxiety and depression can affect executive function and attention, potentially impairing task completion [17]. However, the majority of participants did not miss deadlines, indicating that faculty may employ effective compensatory strategies to maintain productivity under psychological stress.

Our sample predominantly consisted of male faculty members (59.7%) in mid-career positions, with a mean age of 42.5 years and an average of 3.8 years at their current position. This contrasts slightly with prior studies that reported longer tenure averages [18], suggesting that even relatively early-stage faculty are experiencing significant psychological burden yet maintaining performance. These mid-career academics may face unique occupational stressors, including increased workload, rapid adaptation to new teaching modalities, and research disruptions, but their reported academic outcomes reflect strong adaptive capacity. Nevertheless, the prevalence of moderate to severe symptoms indicates a potential risk for cumulative stress and burnout, underscoring the need for longitudinal monitoring.

The findings have implications for institutional policy and faculty support. Universities should recognize the resilience of their staff but also proactively provide coordinated mental health interventions to preserve wellbeing and sustain productivity. Prior studies, such as White Berheide et al. [19], emphasize that providing faculty with adequate time, resources, and supportive structures during crises can protect mental health and optimize performance. Interventions such as mindfulness training or cognitive-behavioral strategies may help improve concentration and mitigate emotional disruptions, particularly given the observed associations between depressive symptoms and academic output.

## Conclusion

The study offers insights into the effects of COVID-19 on mental health and performance of the medical faculty at KAU. Despite the mild to moderate anxiety and depressive symptoms caused by COVID-19, most reported a steady academic performance and met academic deadlines. These findings suggest resilience amongst the faculty members to push through despite anxiety caused by the pandemic. Still, there ought to be a system of checks and balances by integrating mental health support in the education sector to enhance excellence. In-depth cross-institutional studies should be conducted to find out the optimal impact of the pandemic on academic performance. The study should also seek to research the effects of the hybrid study on academic performance across multiple institutions.

## Conflict of Interest

There was no conflict of interest regarding the publication of this paper, nor was any external funding offered.

## AI Disclosure Statement

During the preparation of this manuscript, the authors used ChatGPT, OpenAI company for language editing, grammar improvement, and liboberry.com for reference management. After its use, the authors thoroughly reviewed, verified, and revised all AI-assisted content to ensure accuracy and originality. The authors take full responsibility for the integrity and final content of the published article.
